# Practical joint reconstruction of activity and attenuation with autonomous scaling for time-of-flight PET

**DOI:** 10.1088/1361-6560/ab8d75

**Published:** 2020-12-23

**Authors:** Yusheng Li, Samuel Matej, Joel S Karp

**Affiliations:** Department of Radiology, University of Pennsylvania, Philadelphia, PA 19104, United States of America

**Keywords:** joint reconstruction, scaling determination, attenuation correction, time-of-flight (TOF), positron emission tomography (PET)

## Abstract

Recent research has showed that attenuation images can be determined from emission data, jointly with activity images, up to a scaling constant when utilizing the time-of-flight (TOF) information. We aim to develop practical CT-less joint reconstruction for clinical TOF PET scanners to obtain quantitatively accurate activity and attenuation images. In this work, we present a joint reconstruction of activity and attenuation based on MLAA (maximum likelihood reconstruction of attenuation and activity) with autonomous scaling determination and joint TOF scatter estimation from TOF PET data. Our idea for scaling is to use a selected volume of interest (VOI) in a reconstructed attenuation image with known attenuation, e.g. a liver in patient imaging. First, we construct a unit attenuation medium which has a similar, though not necessarily the same, support to the imaged emission object. All detectable LORs intersecting the unit medium have an attenuation factor of *e*^−1^ ≈ 0.3679, i.e. the line integral of linear attenuation coefficients is one. The scaling factor can then be determined from the difference between the reconstructed attenuation image and the known attenuation within the selected VOI normalized by the unit attenuation medium. A four-step iterative joint reconstruction algorithm is developed. In each iteration, (1) first the activity is updated using TOF OSEM from TOF list-mode data; (2) then the attenuation image is updated using XMLTR—a extended MLTR from non-TOF LOR sinograms; (3) a scaling factor is determined based on the selected VOI and both activity and attenuation images are updated using the estimated scaling; and (4) scatter is estimated using TOF single scatter simulation with the jointly reconstructed activity and attenuation images. The performance of joint reconstruction is studied using simulated data from a generic whole-body clinical TOF PET scanner and a long axial FOV research PET scanner as well as 3D experimental data from the PennPET Explorer scanner. We show that the proposed joint reconstruction with proper autonomous scaling provides low bias results comparable to the reference reconstruction with known attenuation.

## 1. Introduction

In positron emission tomography (PET) imaging, attenuation correction with accurate attenuation estimation is crucial for quantitative patient studies. For a modern PET scanner combined with a computed tomography (CT) scanner, the attenuation image is usually estimated from the CT scan by scaling to the PET energy of 511 keV (Kinahan et al 1998). There are situations that the CT-based attenuation is incomplete or inaccurate, due to, e.g. mismatch between PET and CT scans because of patient motion during and between the two scans. PET images reconstructed without attenuation correction, or with incorrect attenuation image can produce incorrect image values and severe artifacts and lead to diagnostic errors (Gould et al 2007). For PET/MR scanners, attenuation image is usually obtained by segmenting the registered MR image with attenuation assignment, or creating a pseudo-CT image by co-registering to a patient’s MR image based on template/atlas information (Martinez-Möller et al 2009, Hofmann et al 2011, Keereman et al 2013, Burgos et al 2014, Ladefoged et al 2017). However, attenuation correction for PET/MR scanners can remain more prone to errors than that for PET-CT scanners since the relation between MR measurements and linear attenuation coefficient at 511 keV is complicated (Wagenknecht et al 2013).

Modern PET systems use very fast gamma-ray detectors and time-to-digital converters to precisely measure the arrival times of the two coincident gammas to provide time-of-flight (TOF) information. As concomitant benefits, TOF information gives a remarkable improvement in image quality of TOF reconstructions, especially in terms of contrast-to-noise ratio (Karp et al 2008, Surti and Karp 2016, Vandenberghe et al 2016, Efthimiou et al 2019). TOF provides localization information of positron annihilation, which improve the robustness of TOF reconstruction in the presence of inconsistent data (Conti 2011). Consequently, TOF PET becomes the de facto standard of modern PET systems for clinical whole-body PET imaging. PET emission data contain attenuation information, and TOF data contain substantially more information about attenuation than non-TOF data. TOF information can be fully characterized by consistency equations (Defrise et al 2013, Li et al 2016b, Li et al 2016a). Applying TOF consistency, Defrise et al (2012) showed that TOF data can determine the attenuation sinogram up to a constant, which results in a multiplicative factor in the activity image. Subsequently, maximum likelihood methods were developed to allow joint reconstruction of attenuation and activity for TOF PET scanners (Salomon et al 2011, Rezaei et al 2012, Rezaei et al 2014, Defrise et al 2014, Nuyts et al 2018). For an extensive overview on the subject of attenuation correction from TOF emission data, there is a detailed review paper by Berker and Li (2016).

Next generation TOF PET scanners using silicon photomultiplier (SiPM) or digital SiPM based detectors have significantly improved timing resolution (Hsu et al 2017, Casey et al 2017, van Sluis et al 2019, Karp et al 2020). The best commercially available timing resolution for current clinical TOF PET scanners is 210 ps (Reddin et al 2018, van Sluis et al 2019). In addition, the extended axial field-of-view of PET scanners can dramatically improve the total sensitivity. For the extreme cases, the United Imaging uExplorer total-body PET/CT scanner and the PennPET Explorer whole-body scanner were developed by the teams in UC Davis and Penn, respectively (Cherry et al 2018, Badawi et al 2019, Karp et al 2020). The dozens of fold of the increased sensitivity can be used clinically to take advantage of high throughput, ultra low-dose imaging, or both. For the ultra low-dose imaging applications, e.g. in pediatric imaging (Schmall et al 2018), attenuation correction using an additional CT scan can introduce unnecessarily radiation dose to patients, especially in the scenarios that attenuation images can be estimated using joint reconstruction from TOF PET data. Another important application for the joint reconstruction are the patient scans involving organ or patient motions (especially for long, dynamic, or longitudinal studies), where the joint reconstruction provides perfectly spatially aligned emission and transmission images for each temporal frame. Both the superior timing resolution of the modern PET scanner and the dramatically increased sensitivity from the long axial FOV scanners can be beneficial to the joint reconstruction by providing increased Fisher information and improved noise performance, which also motivated this work. The long axial FOV scanner is also challenging for joint reconstruction due to the large acceptance angles of acquired LORs with large variation of normalization and attenuation factors.

The TOF information help to disentangle between activity and attention and make joint reconstruction of activity and attenuation possible. The rationale behind joint reconstruction is that TOF can help to localize the origins of annihilation events, and the localized activity source in turn serves as a distributed source at the same energy of 511 keV for estimating transmission attenuation. Utilizing TOF consistency information, attenuation can be determined from emission data, jointly with reconstructed activity images, up to a scaling constant (Defrise et al 2012). Without determining the scaling, joint reconstruction, e.g. MLAA or MLACF, can diverge even starting with the true activity and attenuation images. The scaling can only be determined using external information, which can be a major factor limiting the practicality of joint reconstruction in clinical applications. Theoretically, the scaling can be determined using the boundary condition with TOF consistency equations (Li et al 2015a). In practice, variant methods with different limitations can be used to determine scaling, e.g. using MR information (Salomon et al 2011, Mehranian and Zaidi 2015, Zhu et al 2016, Ahn et al 2018), scattered or single events (Berker et al 2014, Feng et al 2018), or additional transmission sources (Mollet et al 2012, Panin et al 2013).

We aim to develop practical CT-less joint reconstruction for clinical TOF PET scanners to obtain quantitatively accurate activity and attenuation images. In this work, we develop a joint reconstruction of activity and attenuation based on MLAA with autonomous scaling determination and joint TOF scatter estimation at each iteration from TOF data. Our idea is to use a selected volume of interest (VOI) or multiple VOIs in a reconstructed attenuation image with known attenuation, e.g. a liver in patient imaging. First, we construct a unit attenuation medium which has a similar, not necessarily the same, support to the imaging patient. All detectable LORs intersecting the unit medium have an attenuation factor of about *e*^−1^ ≈ 0.3679, i.e. the line integral of attenuation coefficients is one. The scaling factor can then be determined from the difference between the reconstructed attenuation image and the known attenuation within the selected VOI(s) divided by the unit attenuation medium. The scaling determination allows the practical joint reconstruction to obtain a unique and faithful solution of activity and attenuation from TOF PET data. Some preliminary results have been presented at the Fully 3D 2019 meeting (Li et al 2019).

## 2. Joint reconstruction with autonomous scaling

We develop a practical joint reconstruction of activity and attenuation with autonomous scaling and joint scatter estimation. There are in general four steps at each iteration for the proposed joint reconstruction: (1) the activity distribution is updated using the OSEM from TOF list-mode data; (2) the attenuation image is then updated using XMLTR—an extended MLTR—from non-TOF LOR sinograms; (3) the scaling constant is determined based on a selected VOI in attenuation image, then the activity and attenuation images are updated based on the determined scaling constant; and (4) scatter is estimated using TOF single scatter simulation (SSS) with the jointly reconstructed activity and attenuation images. The following two subsections give the algorithms for joint activity and attenuation reconstructions and the method for autonomous scaling.

### 2.1. Joint reconstruction of activity and attenuation

We present a practical joint activity and attenuation reconstruction for time-of-flight (TOF) PET based on MLAA algorithm (Nuyts et al 1999, Rezaei et al 2012). In TOF PET, the expected data ${\bar{y}}_{t}$ for TOF bin *t* can be given by

(1) $${\bar{y}}_{t}=e^{-\chi \mu }H_{t}\lambda +s_{t},$$

where **λ** and ***μ*** are, respectively, 3D distribution of activity and linear attenuation coefficients, $H_{t}$ and *χ* are, respectively, TOF emission and transmission system matrices or operators, *s_t_* is the expected contribution of scatter and randoms. Random events have no TOF information, which can be evenly distributed among all TOF bins within coincidence window. Since the TOF data are Poisson distributed, we write the log-likelihood function after dropping terms independent of ***λ*** and ***μ*** as

(2) $$L\left(y_{t}|\lambda ,\mu \right)=\sum_{t}\left(y_{t}^{\text{T}}\log{\bar{y}}_{t}-1^{\text{T}}{\bar{y}}_{t}\right)$$

Where *y*_*t*_ are the measured TOF data, and **1** is an all-ones vector (all elements are equal to one). There are two sets of unknowns, the activity image and the attenuation image, and it is intractable to simultaneously estimate them by maximizing the log-likelihood function (2). An alternating technique is often used to iteratively maximize the likelihood function—first with fixed attenuation image to update activity image, and then vice versa (Nuyts et al 1999, Rezaei et al 2012). With fixed true attenuation, maximum likelihood expectation-maximization (MLEM) is known to monotonically increase the likelihood. The TOF MLEM is expressed as

(3) $${\hat{\lambda }}^{\left(n+1\right)}=\frac{{\hat{\lambda }}^{\left(n\right)}}{\sum_{t}H_{t}^{\text{T}}a}\sum_{t}H_{t}^{\text{T}}\left[\frac{ay_{t}}{aH_{t}\lambda^{\left(n\right)}+s_{t}}\right],a=e^{-\chi {\hat{\mu }}^{\left(n\right)}},$$

where ***a*** are the attenuation factors in a vector form. For conciseness, we used Hadamard component notations ***ab*** and ***a***/***b*** to represent the componentwise multiplication and division, respectively (Barrett et al 1994, Li 2011).

TOF information can be used to substantially improve the image quality of activity reconstruction, but it does not directly improve attenuation reconstruction^1^. In ideal cases ***s_t_*** = 0, TOF information does not contribute to attenuation based on the log-likelihood function (2). The non-TOF data are used for attenuation reconstruction, which also has efficient storage in binned format. By summing over the TOF bin *t*, the corresponding non-TOF data can be obtained, e.g. $y=\sum_{t}y_{t},s=\sum_{t}s_{t}$, and the non-TOF emission system matrix is $H=\sum_{t}H_{t}$

For attenuation reconstruction with fixed activity image, a similar method to the grouped-coordinate ascent algorithm (Fessler et al 1997), was used to maximize the non-TOF version of the log-likelihood (2).

The update equation for attenuation image is (see appendix A for the detailed derivation)

(4) $${\hat{\mu }}^{\left(k+1\right)}={\hat{\mu }}^{\left(k\right)}+\frac{\chi^{\text{T}}\left[pe^{-\chi {\hat{\mu }}^{\left(k\right)}}\left(1-\frac{y}{pe^{-\chi {\hat{\mu }}^{\left(k\right)}}+s}\right)\right]}{\chi^{\text{T}}\left[pe^{-\chi {\hat{\mu }}^{\left(k\right)}}\left(1-\frac{ys}{{\left(pe^{-\chi {\hat{\mu }}^{\left(k\right)}}+s\right)}^{2}}\right)\chi 1\right]},p=H\lambda^{\left(k\right)}$$

where ***p*** is the activity projection in a vector form. In computed tomography, ***p*** corresponds to the measurements from a blank scan. In the case $y\approx pe^{-\chi {\hat{\mu }}^{\left(k\right)}}+s$, which holds approximately after a few iterations, (4) becomes the MLTR

(5) $${\hat{\mu }}^{\left(k+1\right)}={\hat{\mu }}^{\left(k\right)}+\frac{\chi^{\text{T}}\left[pe^{-\chi {\hat{\mu }}^{\left(k\right)}}\left(1-\frac{y}{pe^{-\chi {\hat{\mu }}^{\left(k\right)}}+s}\right)\right]}{\chi^{\text{T}}\left[\frac{{\left(pe^{-\chi {\hat{\mu }}^{\left(k\right)}}\right)}^{2}}{pe^{-\chi {\hat{\mu }}^{\left(k\right)}}+s}\chi 1\right]},p=H\lambda^{\left(k\right)}.$$

Equations (4) and (5) are similar in terms of maximizing the log-likelihood function. The only practical difference is in the denominator at earlier iterations, and the results using (4) and (5) are numerically similar after a few iterations. When **s** = 0, (4) and (5) become identical. Equation (5) was likely based on an empirical equation (Nuyts et al 1999, Rezaei et al 2012). However, (4) is derivated based on mathematically rigorous formulation, and we refer (4) as XMLTR—an extended MLTR update equation.

### 2.2. Scaling determination

It has been shown by Defrise *et al* that TOF data can determine the attenuation factor up to a constant (Defrise et al 2012). This undetermined constant can cause severe problems for practical applications of the joint activity and attenuation reconstruction. After finding one special solution to (1), e.g. *λ*_0_, *μ*_0_, the general solution is

(6) $$\lambda =C\lambda_{0},\mu =\mu_{0}+\mu_{\text{add}}$$

where *C* is multiplicative constant to activity image, and *μ*_add_ is an additive attenuation medium that produces an attenuation factor of *C*^−1^ for each LOR, i.e. $e^{-\chi \mu_{\text{add}}}=C^{-1}$. The jointly reconstructed activity and attenuation images from MLAA can be considered as one special solution, and we need to determine the scaling *C* to obtain the true activity and attenuation.

For practical imaging applications, *λ* and *μ* both have finite support. The support can be determined, e.g. by finding a convex region outside of where there is no or little activity from activity images reconstructed without attenuation correction, by back-projecting and thresholding operations (Nuyts et al 1999), or can be synthesized from reconstructed images, or even obtained using 3D scanning or machine learning techniques. Usually, it is not difficult to find a volume of interest (VOI) in attenuation image *μ* to have known attenuation, e.g. a liver region with fairly uniform linear attenuation coefficients. We can use the selected VOI and determine the scaling constant by examining the reconstructed attenuation image in this VOI. First, we introduce a unit attenuation medium, which produce a unit line integral of attenuation,

(7) $$\chi \mu_{\text{unit}}=1_{\Omega }$$

where Ω denotes the support of attenuation of the whole object in sinogram or data space, and **1**_Ω_ is an indicator vector (equal to one within Ω, and zero otherwise). Ideally, all LORs intersecting with Ω, the attenuation factor produced by *μ*_unit_ is *e*^−1^≈ 0.367 9. A simple 2D example of unit attenuation medium in a disk region with radius *R* is given by $\frac{1}{\pi \sqrt{R^{2}-r^{2}}}\prod \left(\frac{r}{2\text{R}}\right)$. Here, *r* is the distance to origin, and ∏ (*x*) is the rectangular function which is one for |*x*| < 1/2 and zero otherwise. This unit attenuation medium is singular at the boundary of the disk (Defrise et al 2012). So voxels near boundaries should be avoided when selecting VOIs with known attenuation. In general, the exact solution in 3D to (7) with different supports may not exist; however, one can always compute an approximated solution numerically for practical imaging applications. Examples of such unit attenuation medium computed for particular reconstructed objects considered in this work are illustrated in the respective results sections. By comparing *μ*_add_ and *μ*_unit_ within the VOI with known attenuation, we can determine the scaling factor at (*k* + 1 )th iteration as

(8) $$\log C^{\left(k+1\right)}=\frac{w_{\text{voi}}^{\text{T}}\mu_{\text{true}}-w_{\text{voi}}^{\text{T}}{\hat{\mu }}^{\left(k+1\right)}}{w_{\text{voi}}^{\text{T}}\mu_{\text{unit}}}$$

where ***w***_voi_ denotes the mask (or weighting) for the selected VOI with known attenuation. As the name of *unit medium* suggested—***μ***_unit_ serves as a unit measure—a dimensionless quantity of scaling can be obtained by dividing the attenuation difference and ***μ***_unit_ (the unit of attenuation is cm^−1^). As ${\hat{\mu }}^{\left(k+1\right)}$ converging to λ_true_, $\log C^{\left(k+1\right)}\to 0$, the scaling iteratively approaches to one, and the final attenuation reconstruction is independent of the unit medium ***μ***_unit_. Once the scaling constant at current iteration is determined, we can use it to update activity and attenuation images as follows:

(9) $${\hat{\lambda }}^{\left(n+1\right)}\leftarrow C^{\left(k+1\right)}{\hat{\lambda }}^{\left(n+1\right)}$$

(10) $${\hat{\mu }}^{\left(k+1\right)}\leftarrow {\hat{\mu }}^{\left(k+1\right)}+\log C^{\left(k+1\right)}\mu_{\text{unit}}$$

After the updates in (9) and (10), the reconstructed attenuation image has matched attenuation with the true attenuation within the selected VOI. The corresponding activity image is also updated to give a better estimate of initial image for the next iteration of activity update. Due to the robustness of algorithms (3) and (4), only one update using either (9) or (10) might be enough for convergence. We use both updates (9) and (10) for a smooth updating between iterations and for fast convergence using the information of computed unit attenuation medium and scaling constant. The additive attenuation $C^{\left(k+1\right)}\mu_{\text{unit}}$ in (10) can be negative when the scaling factor is less than one. It is reasonable to constrain the reconstructed attenuation image with non-negative values at each iteration.

## 3. Simulations and experiments

### 3.1. 3D simulations of a generic TOF PET scanner

**Figure 1.**
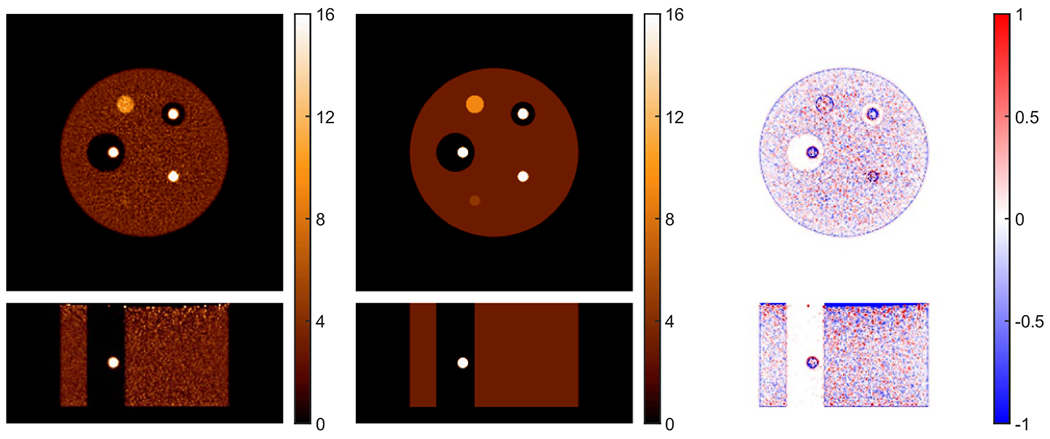
Comparison of reference activity reconstruction (left) using 3D TOF OSEM and true activity image (middle) for the 3D simulation. The difference between reconstructed image and the true activity image normalized by the average background is also shown (right). The voxel size for the reconstructed activity image is 2 mm with image size of 288 × 288 × 125. The transverse and coronal images are respectively shown in top and bottom. The activity phantom comprises a uniform cylinder with diameter of 35 cm and hot spheres of 22 and 37 mm with different contrasts.

We performed EGS4 based Monte Carlo simulations for a generic clinical TOF PET system with timing resolution of 300 ps. The scanner has a diameter of 84.2 cm and an axial field-of-view (FOV) of 25 cm. There were 616 × 58 LSO crystals of 4 × 4 × 20 mm^3^ along transverse and axial directions. We have designed a dedicated phantom for the evaluation of joint reconstructions. The activity phantom comprises a uniform cylinder with diameter of 35 cm and hot spheres of 22 mm with contrasts 1.5 and 6, and 37 mm with contrast 3 at different locations, as shown in figure 1. The scattered and random events were not included. The attenuation phantom has mostly the same attenuation as that of the water, 0.096 cm^−1^ at 511 keV. Two uniform cylinders located at (—8,0,0) cm and (6,8,0) cm with diameters of 8 and 5 cm have attenuation coefficient of one-third of water and zero to represent lung and air, respectively. Normalization sinograms were generated by simulating a uniform cylinder phantom of 40 cm diameter with a very large number of coincident events. The normalization correction factors were then obtained by computing the ratio of the simulated sinogram to an analytical sinogram from the uniform cylinder phantom.

### 3.2. 3D simulations of the PennPET Explorer scanner

We also performed GATE simulations for a long axial FOV research PET scanner based on the PennPET Explorer scanner (Viswanath et al 2017). While the long axial FOV scanner leads to high sensitivity, it also represents a challenge for the joint reconstruction due to the fact that it contains oblique LORs with large acceptance angles and with large variations of normalization and attenuation factors. We believe this geometry thus provides good test of the robustness of the scaling estimation in the joint reconstruction studied. The scanner has a diameter of 76.4 cm and an axial field-of-view (FOV) of about 70 cm. The PennPET Explorer comprises three ring segments, each segment of 23.0 cm axial length consists of 18 modules with 5 × 7 detector tiles of 3.86 × 3.86 × 19 mm^3^ LYSO crystals (Karp et al 2020). The timing resolution is 250 ps. We converted the GATE output ROOT file into list-mode file format including both true and scattered events. We used the anthropomorphic XCAT phantom with an adult female configuration (Segars et al 2010). The organ regions were filled to reflect activity concentrations 1 h after an injection of 10 mCi FDG. SUV values were determined for each organ of interest based on both literature (Ramos et al 2001, Wang et al 2007), and measurements on clinical FDG patients imaged at the University of Pennsylvania. The average concentrations were 1.51, 2.41, 3.02, 4.52, 7.24, 7.84, 9.05, 9.65, 12.07 and 24.13 kBqcm^−3^ for body/fat, lung, breast, muscle, pancreas, gut, blood pool, spine, liver/kidney, myocardium, respectively. The use of XCAT phantom is to test the scaling method on a more challenging object with realistic organ shapes and attenuation and for a challenging PET geometry with long axial FOV. Geometric normalizations were generated by simulating a 60 × 70 cm non-attenuating cylinder filled with 1 mCi activity. The size of the normalization sinogram was 321 × 288 × 156 × 156.

The image reconstruction for the PennPET Explorer scanner with large data sets is challenging, let alone joint reconstruction of activity and attenuation. Similar to the data corrections used for our list-mode TOF reconstruction, normalization and delayed randoms correction were included during joint reconstructions of both activity and attenuation. For the details on data corrections, please refer to the recent publication on the design and preliminary performance of the PennPET Explorer (Karp et al 2020). For scatter correction, scattered events were estimated based on TOF enhanced single scatter simulation (SSS) method (Accorsi et al 2004, Werner et al 2006, Watson 2007). TOF scattered events were estimated using jointly reconstructed activity and attenuation images after being updated based on the estimated scaling using (9) and (10) at each iteration. Then scatter corrections were applied to both activity reconstruction and attenuation reconstruction using TOF-OSEM and XMLTR, respectively.

### 3.3. 3D experiments using the PennPET Explorer scanner

We further evaluated the joint reconstruction with autonomous scaling using 3D experimental data from the PennPET Explorer scanner (Karp et al 2020). The coincidence events are collected within each ring segment and between any ring combinations. Due to a limit in current read-out, there was an inter-ring axial gap of 7.56 cm in the collected data. The active axial FOV was 64 cm instead of 70 cm; however, these data with gaps allowed us to experimentally test the joint reconstruction in the challenging case with axial gaps. With optimized trigger level setting, the PennPET Explorer has timing resolution of 250 ps. The same scatter estimation method in simulations was used for the experiments.

A SNMMI Clinical Trials Network (CTN) oncology phantom was scanned on the PennPET Explorer scanner (Ulrich et al 2018). The CTN phantom has an axial length of 30 cm, which falls short compared with the axial FOV. A uniform cylinder phantom was attached to the CTN phantom to fully utilized the axial FOV. The CTN phantom has similar spherical inserts as the NEMA IEC phantom ranging from 10 mm to 37 mm. The data were collected in list-mode format, and correction data, e.g. normalization, random correction, are stored in LOR sinogram (Kadrmas 2004, Li et al 2014). The size of the sinogram is 301 × 288 × 120 × 120.

## 4. Results

### 4.1. 3D simulations of a generic TOF PET scanner

**Figure 2.**
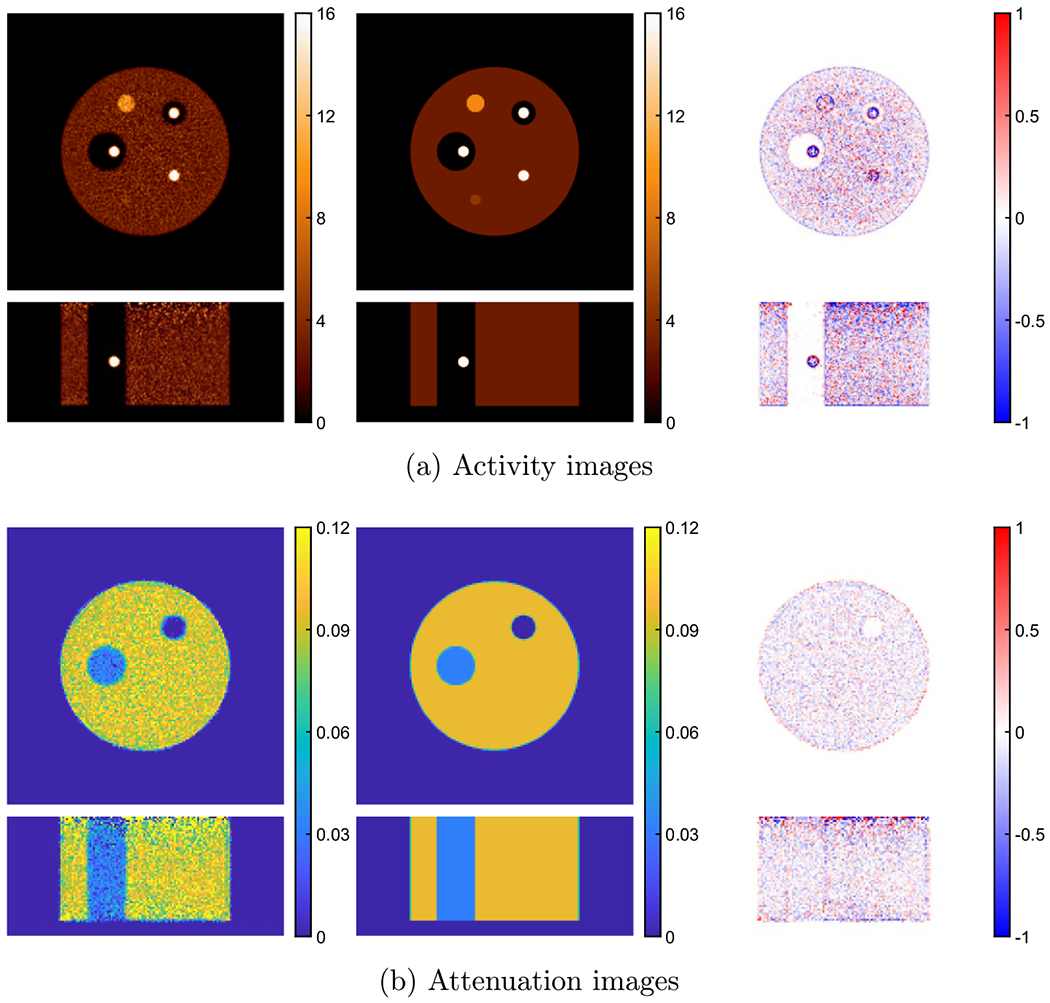
Comparison of joint reconstruction of activity (a) and attenuation (b) with autonomous scaling for the 3D simulation. The reconstructed images, true images and the difference image normalized by the average background are shown in left, middle and right, respectively. The voxel size of the activity image is 2 mm with image size of 288 × 288 × 125, while the voxel size of the attenuation image is 4 mm with image size of 144 × 144 × 62.

Figure 1 shows the reference reconstruction using 3D list-mode TOF OSEM with attenuation correction from the true attenuation image. The true activity image and the difference image normalized to the background value are also shown. The true attenuation image that was used for attenuation correction is shown in figure 2(b). There were about 100 × 10^6^ total true coincidence events from the EGS4 simulation without scatter and randoms. We deliberately used a large number of counts so that the results would not be dominated by statistics in this initial test. The blob-based instead of voxel-based reconstruction was used for better noise performance, and the generalized Kaiser–Bessel basis function with shape parameter 8.63 was used in the reconstructions (Lewitt 1992, Matej and Lewitt 1995, Matej and Lewitt 1996). And the body-centered cubic (BCC) grid was used, which is proved to have optimal sampling grid based on multidimensional sampling theorem (Li 2018). The BCC grid step and blob radius were 4 and 5 mm, respectively. There were 10 iterations with 25 time subsets. The reconstructed blob images were then converted into voxel images of 288 × 288 × 25 with voxel size of 2 mm for display.

**Figure 3.**
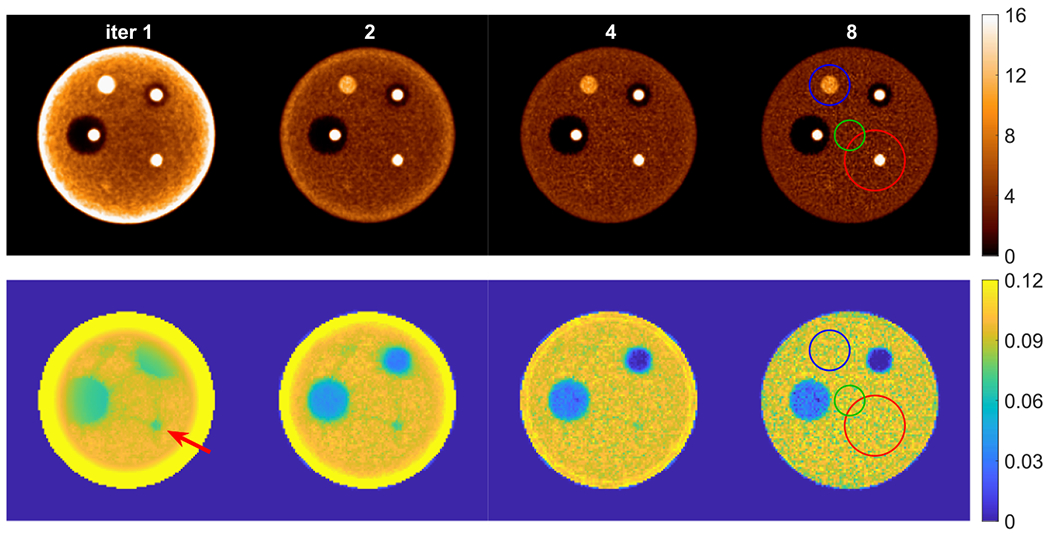
Comparison of joint reconstruction of activity (top) and attenuation images (bottom) at different iterations. The joint reconstruction started with zero attenuation, and the iterations are 1, 2, 4 and 8 from left to right. The bottom arrow shows the crosstalk location between activity and attenuation, and the crosstalk artifacts gradually decrease as the increase of the iteration number. Three different VOIs, VOI-1, VOI-2 and VOI-3, were used for scaling determination, which are respectively indicated by the bottom right (red), central (green) and top left (blue) circles in the right images.

Figure 2 shows the joint reconstruction of activity and attenuation with the VOI-based scaling determined with zero initial attenuation, i.e. no attenuation correction at first iteration of activity reconstruction. The true activity and attenuation images, and the difference images normalized by corresponding background values are also shown. To ensure convergence, there were 10 global iterations, and each global iteration included one iteration of activity update with 25 time subsets, one iteration of attenuation update with 28 geometric subsets, and one step to determine the scaling factor. The same 3D list-mode TOF OSEM was used for activity reconstruction in both joint reconstruction and reference reconstruction. The proposed XMLTR was used for attenuation reconstruction implemented using voxel basis. The size of attenuation is 144 × 144 × 62 with 4 mm voxels. A cylindrical object support (image space mask with non-zero activity) with additional 1 cm margin was used during the joint reconstruction. The margin was introduced for possible tolerance in determining the support; a too small support can introduce image truncation errors in joint reconstruction, while a slightly larger support has minimal impact. The comparison of the central transverse slices of jointly reconstructed activity and attenuation images at iterations 1, 2, 4 and 8 is shown in figure 3. As indicated by the arrow, there is obvious crosstalk in reconstructed attenuation images at earlier iterations; however, the crosstalk artifacts gradually decrease or disappear as the joint reconstruction of activity and attenuation converges after a few iterations.

**Figure 4.**
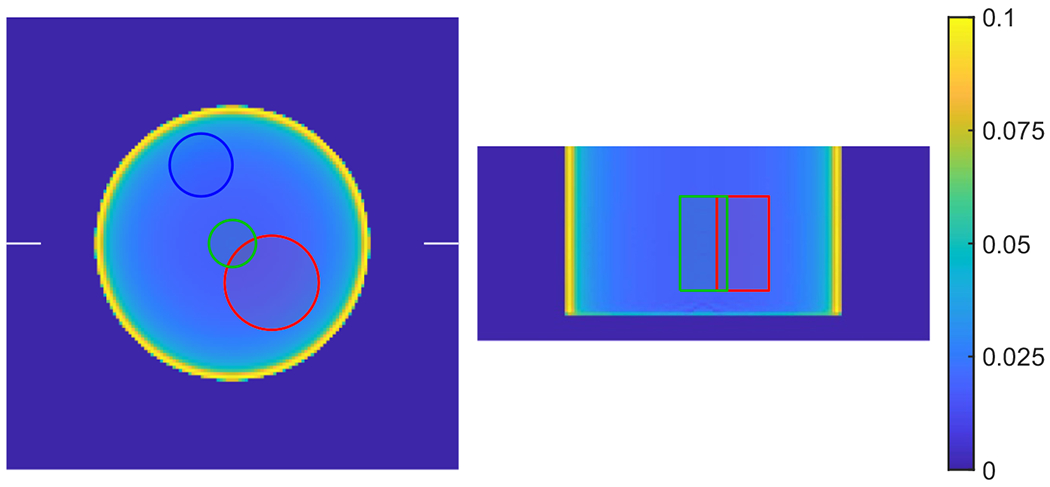
Illustration of the computed unit attenuation medium for the cylindrical phantom, which is required for the autonomous scaling procedure within the joint reconstruction. All detectable LORs intersecting the unit medium have line integral values of one. The circles and rectangles in, respectively, the transverse (left) and coronal (right) views indicate locations of the three different VOIs used for scaling determination.

Before joint reconstruction, unit attenuation medium employed in the autonomous scaling procedure needs to be computed for the particular reconstructed object. To compute the unit attenuation medium, we set the projection values for all LORs intersecting the object support to a constant value of *e*^−1^≈ 0.3679 and one otherwise. We then performed XMLTR reconstruction using these projections to obtain an approximation of the unit attenuation medium; we used five iterations and 28 subsets. Figure 4 shows the computed unit attenuation medium for the cylindrical phantom used in this study. As illustrated, the unit attenuation medium has very high attenuation values near the boundaries and lower values deep inside the object.

**Figure 5.**
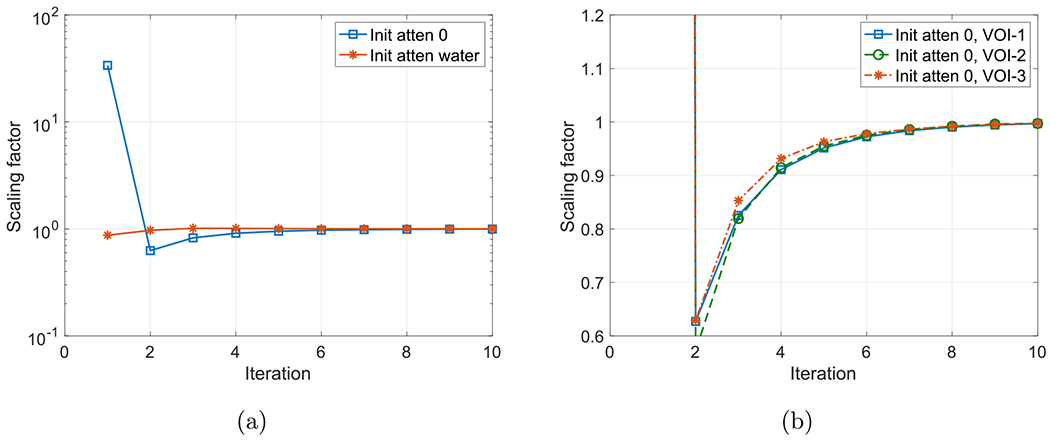
The estimated scaling factors versus iteration numbers for the two cases with different initial attenuation images: zero attenuation and water attenuation (a) and for three different selected VOIs (b).

For scaling determination purpose, we pre-selected a cylindrical VOI (VOI-1) located at (5, −5, 0) cm within the object with diameter and length of 12 and 12 cm, respectively, to determine scaling during joint reconstruction using (8) at each iteration. We also selected two additional VOIs at different locations to investigate whether the location of VOI affects the scaling estimation. The axial length of the two VOIs were the same as VOI-1. The locations were (0, 0, 0) cm and (–4, 10, 0) cm, and the diameters were 6 and 8 cm for VOI-2 and VOI-3, respectively. The cylindrical VOIs are indicated by the circle and rectangle in the transverse and coronal views in figures 3 and 4. Inside the VOIs, the true attenuation is constant, while the activity may or may not contain lesions and the unit attenuation medium has varying attenuation. To investigate the convergence of the scaling factors, we run joint reconstruction with two different initial attenuations and three different VOIs. We used zero attenuation, i.e. no attenuation correction for activity reconstruction at the first iteration, and a uniform attenuation of water within the phantom support as the initial attenuation images. The scaling factors versus iteration numbers for the two cases with different initial attenuation and for three different selected VOIs are shown in figures 5(a) and (b), respectively. The estimate scaling factors have different converging curves with different initial attenuation images. Even for the zero initial attenuation, the scaling factor converges to unity after a few iterations. The locations and sizes of VOIs has little impact on the scaling estimation.

**Figure 6.**
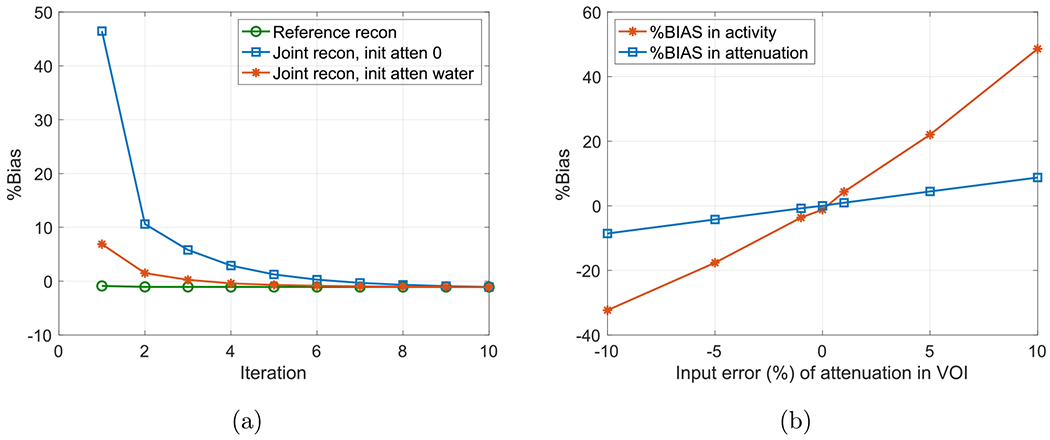
(a) Comparison of %Bias of reconstructed images from the reference reconstruction and joint reconstructions with different initial attenuations. (b) Robustness test of autonomous scaling method with different input error (%) of the average attenuation in VOI.

For a quantitative comparison, we calculated the %Bias and contrast recovery coefficient (CRC) versus background variability to compare the joint reconstruction and reference reconstruction with CT attenuation (Daube-Witherspoon et al 2012, Li et al 2015b). We calculated the %Bias in the cylindrical VOI free of lesions at (2, 1, 0) cm with diameter and length of 8 and 12 cm, respectively. Figure 6(a) shows the comparison of the calculated %Bias, which is normalized to the true activity image. We also computed the %Bias in the whole image excluding the slices on both ends, and the %Bias for the joint and reference reconstructions were within 1% after iterations. The same cylindrical VOI for %Bias was used to calculate the background variability. A local spherical shell region of 4 mm thick surrounding each lesion with 4 mm clearance was used to calculate local background activity concentration for calculating the CRCs. For robustness test of the autonomous scaling, we deliberately injected errors in the average attenuation in the selected VOI with known attenuation, i.e. *μ*_true_ in (8). We performed joint reconstructions with the proposed scaling method with both under- and over-estimation errors in VOI-I. The input errors were ± 1%, ± 5%, ± 10% of the average attenuation in the VOI. Figure 6(b) shows the computed %Bias versus the input error of attenuation for the joint reconstruction. The %Bias in the reconstructed activity image is more sensitive to the input errors than that in the reconstructed attenuation image, which is theoretically reflected by the fact that attenuation factors are exponentially related to the linear attenuation coefficients.

**Figure 7.**
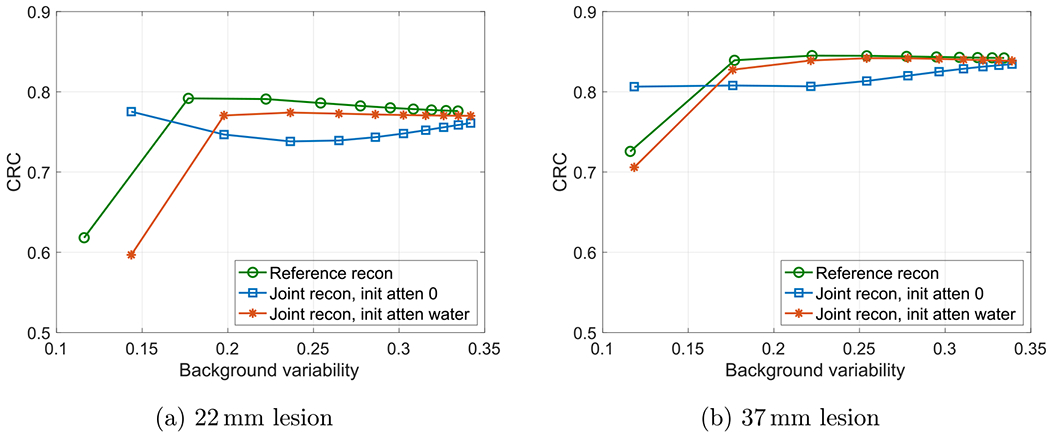
Comparison of CRC versus background variability for the reference reconstruction and joint reconstructions with different initial attenuations.

Figure 7 shows the comparison of CRC versus background variability of the 22 and 37 mm lesions in warm background for the reference reconstruction and joint reconstructions with different initial attenuations. The joint reconstructions with different initial attenuations can have very different CRC versus background variability curves at earlier iterations. The joint reconstruction with zero initial attenuation had unintended large CRCs at earlier iterations since the reconstructed attenuation image used for attenuation correction were not fully converged at these iterations (see the image at iteration 2 in figure 3).

### 4.2. 3D simulations of the PennPET Explorer scanner

**Figure 8.**
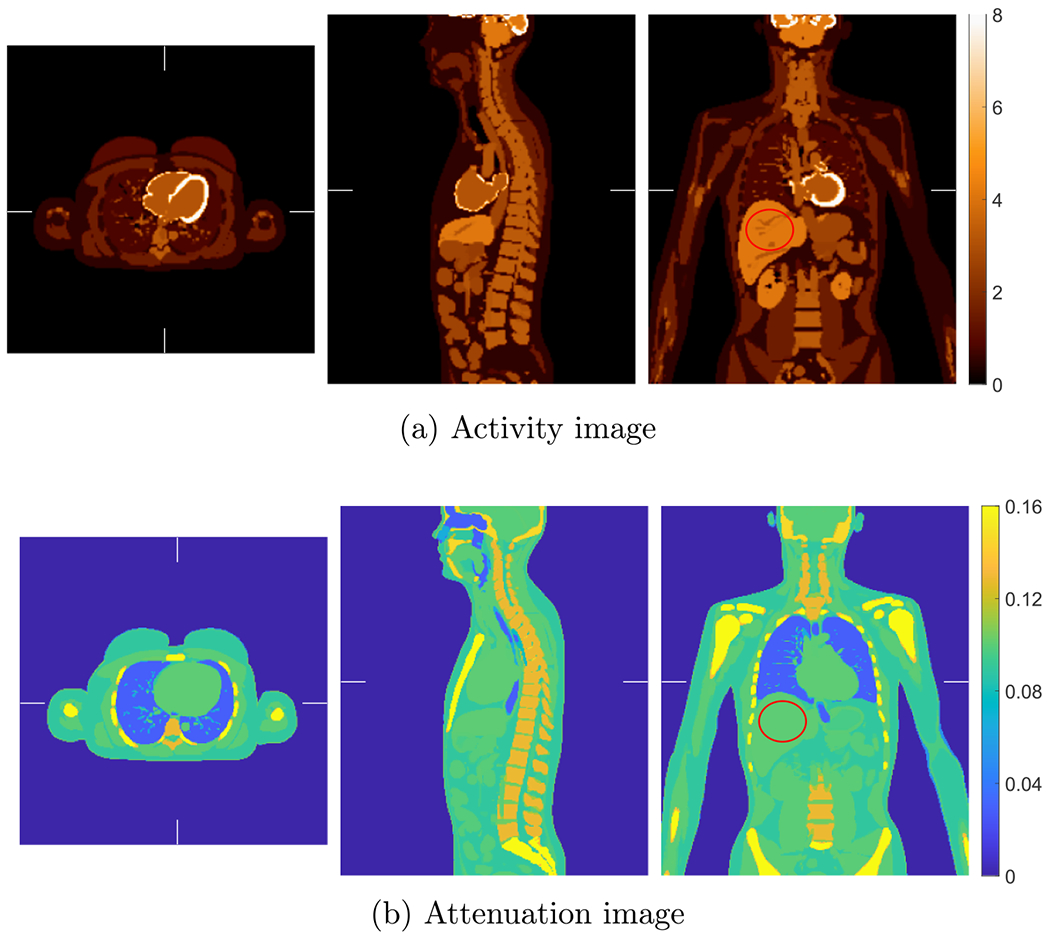
The true activity and attenuation images of the XCAT phantom with transverse, sagittal and coronal views shown from left to right, respectively. The circle in the coronal image indicates the selected VOI in liver for scaling determination.

**Figure 9.**
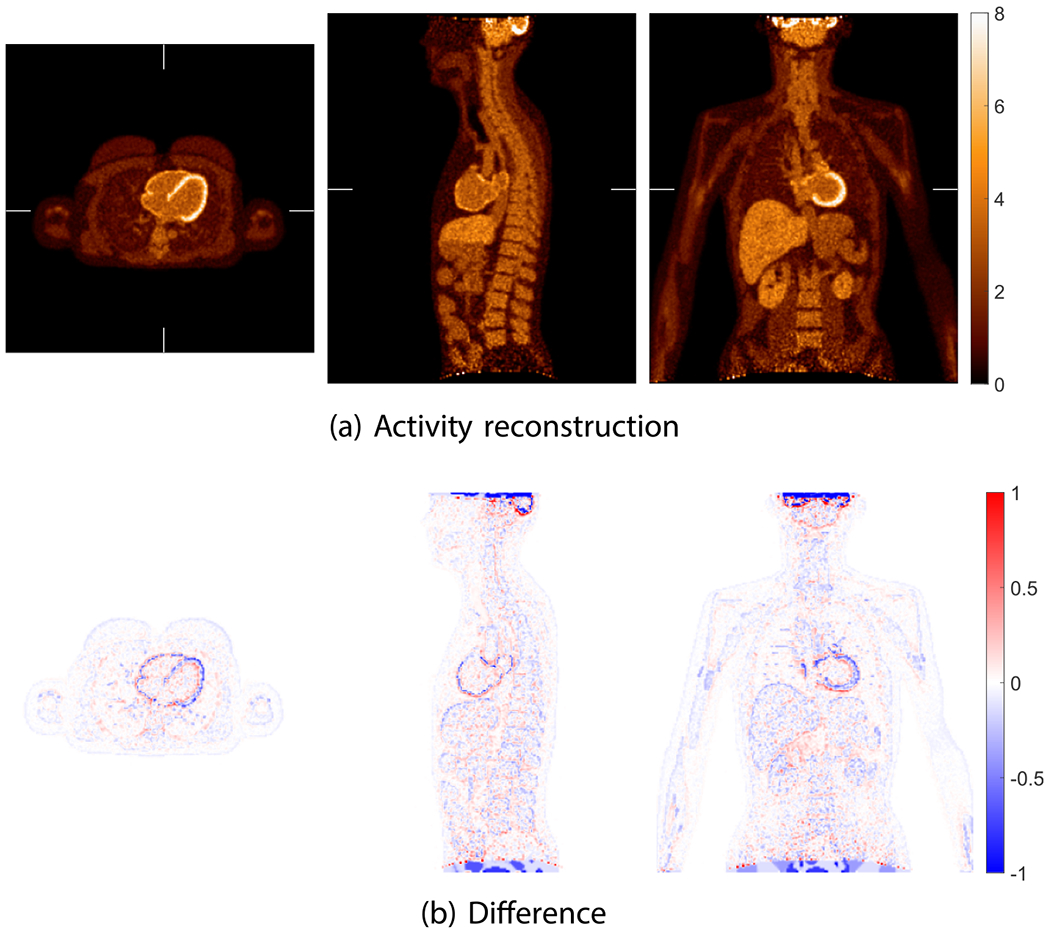
The reference activity reconstruction using TOF OSEM with attenuation correction using the true attenuation image from simulated data with XCAT phantom. The difference image between reconstructed activity image and true activity image normalized by the background value is shown in (b). The voxel size for both activity image and attenuation image is 2 mm.

Figure 8 shows the true activity and attenuation images of the XCAT phantom with voxel size of 2 mm. Figure 9 shows the reference activity reconstruction with attenuation using the true attenuation image and the difference between the reference reconstruction and true activity image normalized using the background value. The background value was calculate in an ellipsoidal VOI insider liver indicated by the ellipse in figure 8. We used the blob-based 3D list-mode TOF OSEM for activity reconstruction and XMLTR for attenuation. The true and scattered events were, respectively, 460 × 10^6^ and 190 × 10^6^ in the 3 min scan. For the reference reconstruction, the scatter was estimated using TOF SSS from the reference reconstructed activity image and the CT attenuation image. Due to the smooth nature of scatter distribution, a coarse grid with downsampled detector crystals was used in TOF SSS based on our routine scatter estimation (Werner et al 2006). We used 21 axial and 36 transverse downsampled crystals in the scatter simulation. The simulated scatter sinograms were then scaled by tail fitting for each slice. The tail fitting region was specified by a tail sinogram, which is generated by forward projecting the attenuation image after removing bed. There were 4 total iterations for scatter estimation. The details of implementation of the scatter estimation are given in Accorsi et al (2004) for the non-TOF case and updated in Werner et al (2006) for the TOF case. Then we performed the reference reconstruction using the estimated scatter and know attenuation. There were 10 iterations with 25 time subsets, and the reconstructed activity image shown in figure 9 is the 5th iteration. The BCC grid sampling and generalized Kaiser–Bessel blob were used; the grid step and blob radius were 4 and 5 mm, respectively. The reconstructed blob images were then converted into voxel images with size of 288 × 288 × 346 and 2 mm voxels.

**Figure 10.**
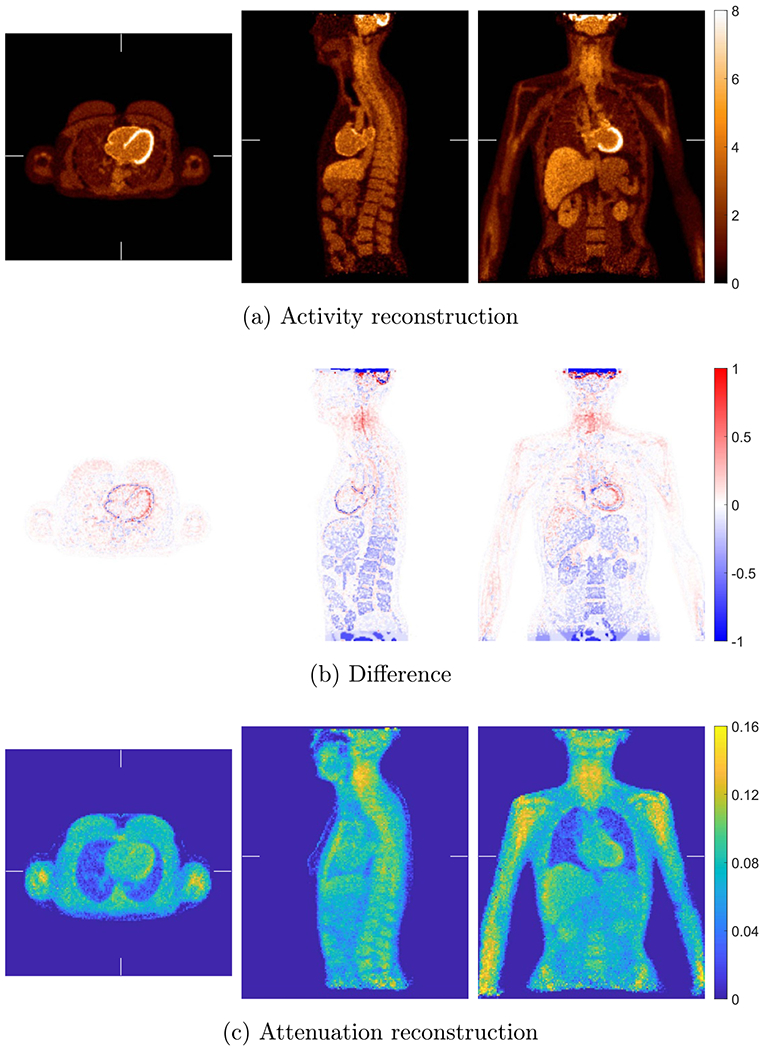
Joint reconstruction of activity (a) and attenuation (c) with autonomous scaling for the simulated data with XCAT. The difference image between reconstructed activity image and true activity image normalized by the background value is shown in (b). The voxel size of the attenuation image is 4 mm, and the voxel size of the activity image is 2 mm with image size of 288 × 288 × 346.

Figure 10 shows the joint activity and attenuation reconstructions with autonomous scaling for the XCAT phantom after five global iterations. The difference image between reconstructed activity image and true activity image normalized by the background value in liver is also shown. The same blob-based TOF OSEM was used for activity image reconstruction as in the reference reconstruction. XMLTR (4) was used for attenuation reconstruction with data corrections including normalization and scatter. We used 4 mm voxels with better noise performance for the reconstructed attenuation image, and the image size is 144 × 144 × 173. We ran up to 10 global iterations to ensure convergence. Each global iteration includes one iteration of updating activity image with 25 time subsets, one iteration of updating attenuation image with 32 geometric subsets, one step of scaling estimation, and one step of TOF scatter estimation. We used an initial attenuation image of uniform water 0.096 cm−1 at 511 keV inside the object support mask. The image mask was obtained by finding a convex region outside of which there is no or little activity. The same parameters for TOF SSS were used in the CT-less joint reconstruction as in the reference reconstruction. The reconstructed attenuation image embedded with the bed attenuation was used in SSS. Using the generated attenuation mask image (excluding bed), we obtain a tail sinogram by forward projecting the attenuation mask filled with water for scatter tail fitting. We observed some discrepancies between joint reconstruction and the reference reconstruction with known attenuation, e.g. residual non-uniformity artifacts in the jointly reconstructed activity image.

**Figure 11.**
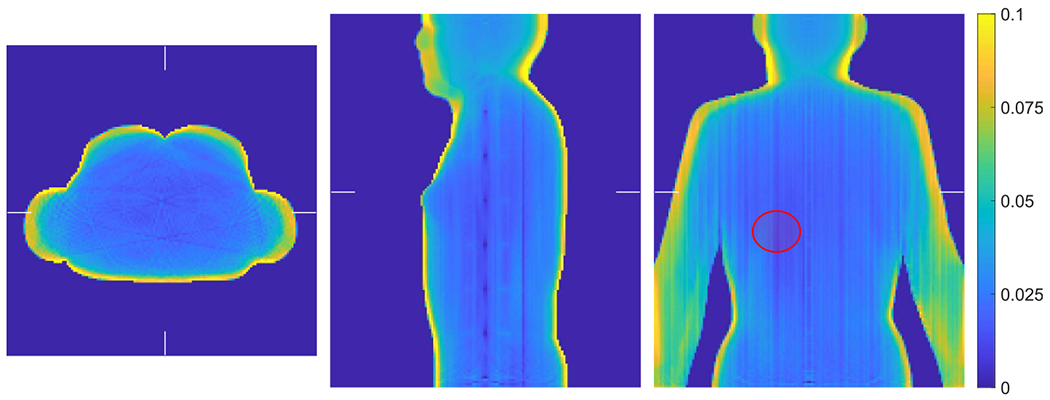
Illustration of the computed unit attenuation medium for the XCAT phantom employed in the autonomous scaling procedure within the joint reconstruction. The images are the transverse, sagittal and coronal views from left to right. The ellipse in the coronal view (right) indicates location of the ellipsoidal VOI, which corresponds to the liver region in the activity image with known attenuation used for scaling determination.

The computed unit attenuation medium employed in the scaling procedure for the XCAT phantom is illustrated in figure 11. An object support Ω in (7) is required for computing the unit attenuation medium, which was generated by forward projecting the object mask in image space. The image support mask had 1 cm margin around the boundaries. We used XMLTR with five iterations and 32 subset to generate the unit attenuation medium for this study. The residual artifacts seen in the computed unit attenuation medium is inconsequential as the scaling factor converges in the joint reconstruction procedure (with the update term in (10) converging to zero).

**Figure 12.**
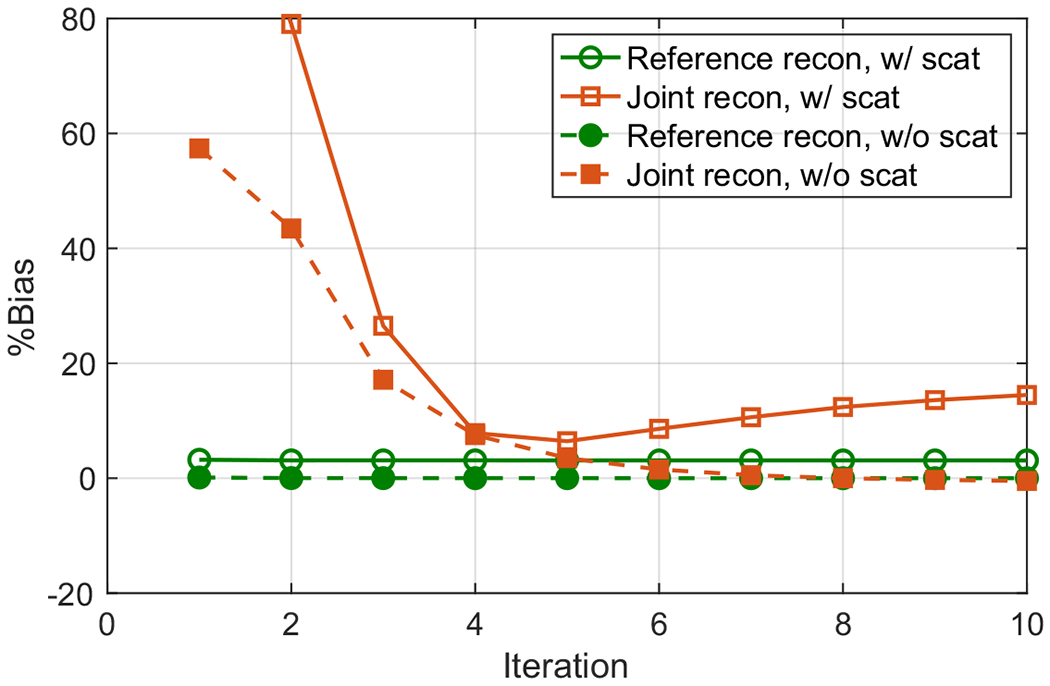
Comparison of %Bias for the reference and joint reconstructions for the XCAT phantom. The filled symbols indicate the results for trues only data, and the open symbols indicate the results for data including scattered events.

For scaling determination in joint reconstruction, we selected an ellipsoidal VOI near the center of the patient liver with, respectively, semi-axes 5.1, 6.3 and 4.5 cm, as indicated in figures 8. We calculated the %Bias normalized by the true activity image in the ellipsoidal VOI to compare the joint reconstruction and reference reconstruction, which is shown in figure 12. The %Bias were 3.1 % and 6.4 % for the reference and joint reconstructions at the 5th iteration, respectively. We also computed the %Bias in the whole image excluding the slices on both ends, and the %Bias were 2.3% and 2.7% for the reference and joint reconstructions at the 5th iteration, respectively. The further increase of %Bias in the joint reconstruction as the increasing of iterations was caused by the discrepancies in scatter corrections. In addition, we performed joint and reference reconstructions using only the true events, and showed that the %Bias did converge to zero consistently for both reconstructions.

### 4.3. 3D experiments using the PennPET Explorer scanner

**Figure 13.**
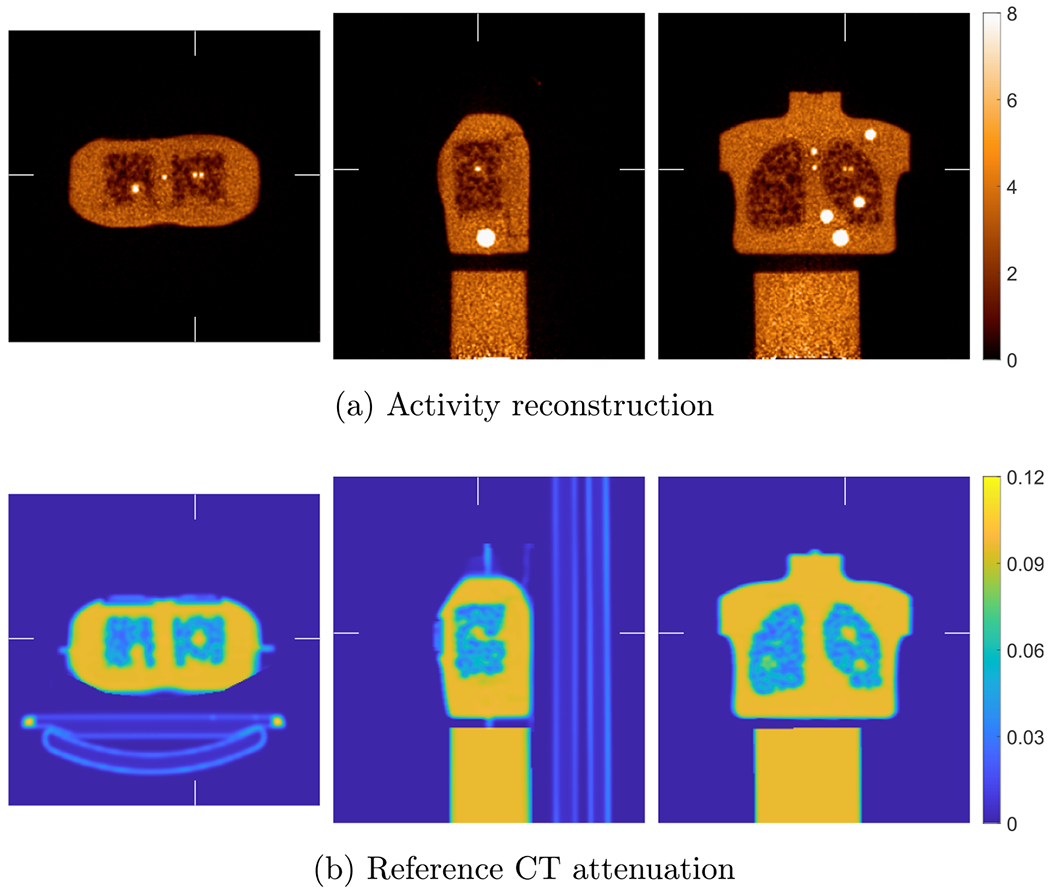
The reference activity reconstruction using TOF OSEM and reference CT attenuation image from experimental data acquired with PennPET Explorer scanner using a CTN phantom attached with a uniform warm phantom. The voxel size for both activity image and attenuation image is 2 mm.

We used the same reference and joint reconstructions as in the simulated reconstructions with XCAT phantom. Figure 13 shows the reference activity reconstruction using CT attenuation correction and the corresponding CT attenuation image. A reference attenuation image was also generated from a CT scan using Philips Ingenuity TF PET/CT with rigid-body registration. The phantom was filled with an activity concentration of 5.9 kBqml^−1^, with lesion to background concentration ratio of about 4:1. The phantom was approximately centered along the axial FOV and was scanned for 60 min, and the data were subsampled to a 6 min scan. The total counts in the 6 min scan were 330 × 10^6^ including scattered events after subtracting the delayed events. The corrections of normalization, attenuation, random, and scatter were applied during the list-mode TOF OSEM reconstruction. We ran up to 10 iterations with 25 time subsets to ensure convergence. The reconstructed activity image shown in figure 13 is the 5th iteration. The same blob based reconstruction was used as for the XCAT phantom, and reconstructed blob images were then converted into voxel images with size of 288 × 288 × 320 and 2 mm voxels.

**Figure 14.**
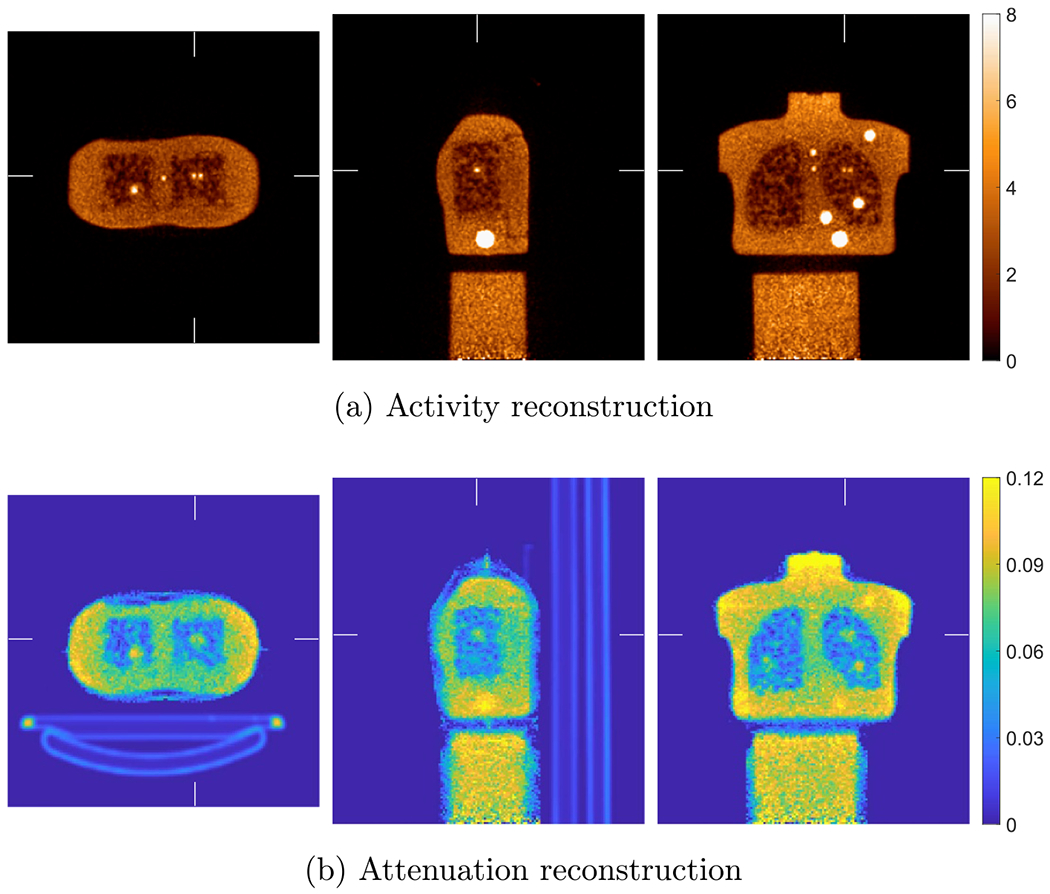
Joint reconstruction of activity (top) and attenuation (bottom) with autonomous scaling for the experimental data acquired with PennPET Explorer using a CTN phantom attached with a uniform phantom. The voxel size of the attenuation image is 4 mm, and the voxel size of the activity image is 2 mm with image size of 288 × 288 × 320.

**Figure 15.**
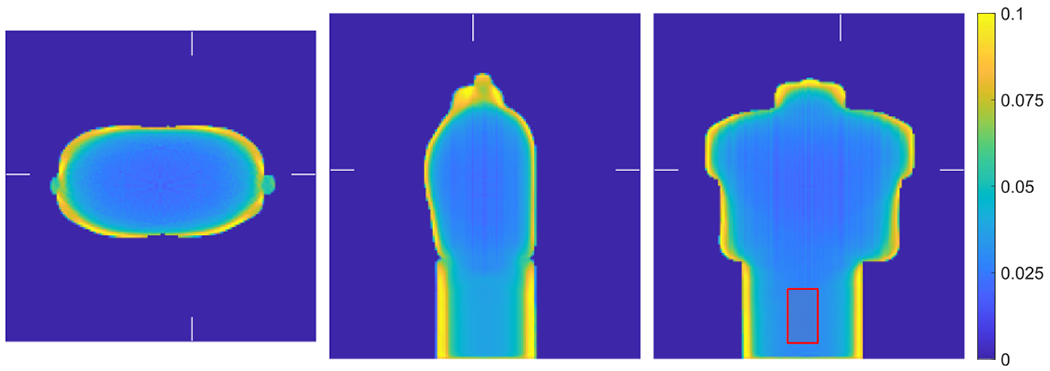
The computed unit attenuation medium for the CTN phantom (with attached uniform cylinder) employed in the autonomous scaling procedure within the joint reconstruction. The images are the transverse, sagittal and coronal views from left to right. The rectangle in the coronal (right) view indicates location of the VOI with known attenuation used for the scaling.

Figure 14 shows the joint activity and attenuation reconstructions with autonomous scaling from the experimental data set after five iterations. We used 4 mm voxels for the reconstructed attenuation image, and the image size is 144 × 144 × 160. We had also included attenuation correction from a patient bed for improved joint reconstructions. The attenuation image of the bed was generated by translating a pre-reconstructed bed attenuation image from a CT scan. The bed attenuation was included in the activity projection $p=H\lambda^{\left(k\right)}$ in (4), the attenuation image without bed was reconstructed using XMLTR. Then the bed attenuation image was embedded into the reconstructed attenuation image. The computed unit attenuation medium employed in the scaling procedure for the CTN phantom (attached with a cylindrical phantom) is illustrated in figure 15. The unit attenuation medium was computed using XMLTR with five iterations and 32 subsets. The attached cylinder phantom has uniform attenuation of water, and for the scaling determination we selected a cylindrical VOI in its center with 12 cm diameter and 10 cm length.

**Figure 16.**
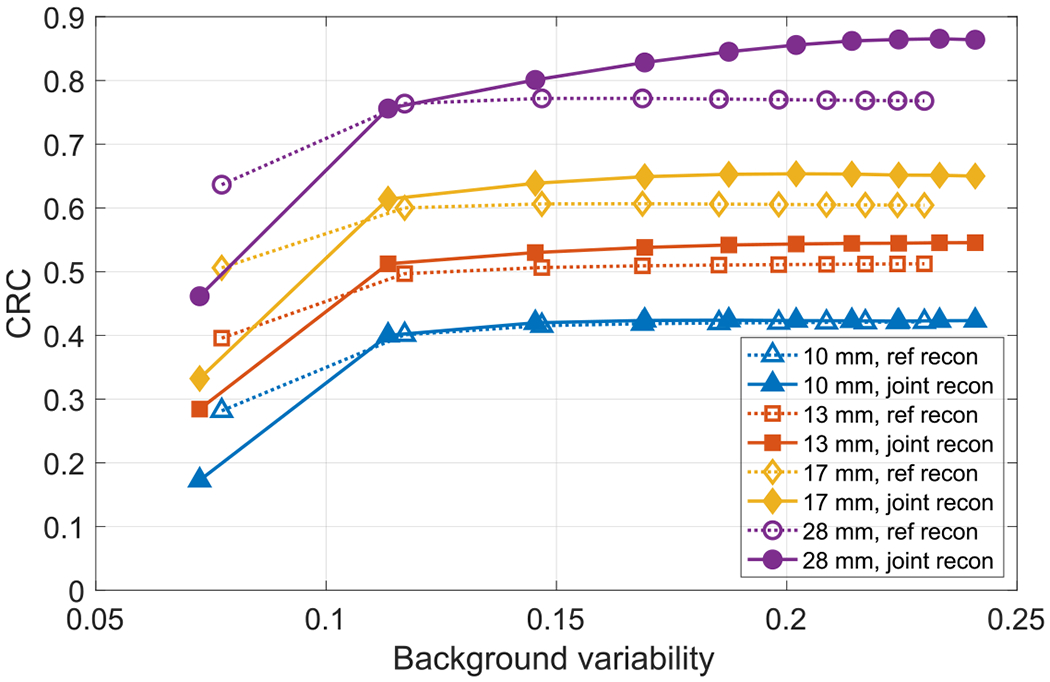
Comparison of CRC versus background variability for the reference reconstruction with CT attenuation and joint reconstruction with autonomous scaling for the experimental data (10 iterations).

We used CRC versus background variability as image quality metric to compare the reconstructions, we calculated the local CRCs for the hot lesions with diameters of 10, 13, 22 and 28 mm. To find the accurate location of the hot lesions, we fitted a local region of reconstructed activity image for each hot lesion using a model of 3D sphere convolved with 3D Gaussian kernel. A local spherical shell region surrounding each lesion was used to calculate local background activity concentration. The thickness of the shell of background activity was 4 mm, and there was a separation of 4 mm between the shell and hot lesion. The background variability was calculated inside the selected cylindrical VOI shown in figure 15. We compared the average values in the cylindrical VOI for the joint and reference reconstructions, and we observed there was about 10% residual difference in this experimental study. Figure 16 shows the comparison of CRC versus background variability of the different sizes of hot lesions for the reference reconstruction and joint reconstruction. The reconstructed activity images from our CT-less joint reconstruction with proper data corrections has comparable performance in terms of CRC versus local background variability compared with the reference reconstruction with CT attenuation. The CRCs for joint reconstruction continue to increase with iteration, especially for the 28 mm lesion, which can be caused by the slightly decreased local background activity.

## 5. Discussion

Time-of-flight PET emission data contain rich information, which allows the possible CT-less joint reconstruction of activity and attenuation. One major challenge is that the TOF consistency information cannot determine the scaling between activity image and attenuation factors (Defrise et al 2012). In this paper, we proposed a novel method to determine the scaling using a selected VOI with known attenuation in average, which make the joint reconstruction of activity and attenuation practical. The proposed method provides a quantitatively accurate scaling between emission and attenuation factors for MLAA based joint reconstructions. The joint reconstruction can find applications when some kind of attenuation related knowledge is available, e.g. a liver with fairly uniform linear attenuation coefficients. The location of VOI in the reconstructed image with a prior known attenuation (e.g. liver) can be obtained without the need of an external scan, e.g. from a preliminary activity reconstruction without attenuation correction, or from a preliminary joint reconstruction without scaling. The scaling determination using (8) does not require a selected VOI having uniform attenuation coefficients nor the knowledge of attenuation coefficient for each voxel inside the VOI, but essentially requires the knowledge of the averaging attenuation within the VOI. As shown in the uniform phantom with true events, the scaling determination in general does not depend on the VOI location as long as voxels near boundaries are avoided. On the other hand, providing accurate attenuation value within the VOI plays an important role in the scaling determination for bias-free reconstructions, as demonstrated in figure 6(b). The scaling determination method can also be generalized. Instead of finding a VOI with known average attenuation, one can find a VOI with stable distribution of linear attenuation coefficients. The scaling factor can then be determined by matching between the attenuation distributions within the selected VOI. We include one remark regarding the bed attenuation. As showed in Defrise et al (2012), Li et al (2015a), the attenuation factor can theoretically be determined provided that there are at least two point sources along the LOR. Unfortunately the bed doest not contain any activity, and most of the LORs passing through the bed have no activity. Therefore the bed attenuation cannot be properly reconstructed within the joint reconstruction and its known attenuation thus cannot be used for the scaling purposes.

Previously, it was shown that the proper determination of the attenuation along an LOR requires that the LOR has at least two points having activity (Li et al 2015a). The LORs are outside of, or tangent to patients or activity regions have zero and one point having activity, respectively. So the recovery of attenuation near the boundaries of patients or outside can be unstable. We suggest to use a patient mask/support (activity is contained within the object support), and attenuation is estimated within the support. In our implementation, the mask was automatically determined by thresholding the projection of reconstructed activity image at each iteration when no mask is provided. Without the mask, the reconstructed attenuation image from joint reconstruction can still provide correct attenuation factor for the non-zero LORs; however, the reconstructed attenuation is not quantitatively accurate. For practical imaging applications, ***λ*** and ***μ*** both have finite support. The support can be determined, e.g. finding a convex region outside of which there is no or little activity from the reconstructed activity images without attenuation correction. Other methods are also possible, e.g. a method using backprojecting and thresholding operations (Nuyts et al 1999), or even using 3D scanning or machine learning techniques. The determined support can in turn be used to determine the unit attenuation medium.

The attenuation is determined within the object support, this is rarely a problem except for the patient table/bed. We also included bed attenuation corrections. During the attenuation reconstruction using XMLTR, the bed attenuation was included in the activity projection ***p*** = ***Hλ***^(*k*)^ in (4), the attenuation image without bed was reconstructed using XMLTR. Then the bed attenuation image was embedded into the reconstructed attenuation image. Since the patient bed usually have approximately translational symmetry along axial direction, the bed position can be determined by measuring the bed height. A bed attenuation image can be generated by translating a pre-reconstructed bed attenuation image from a CT scan based on the bed position. Then, the bed attenuation can be obtain by forward projecting the bed attenuation image.

The joint reconstruction was reconstructed from TOF data acquired from the PennPET Explorer scanner in its prototype configuration with 64 cm axial FOV. Though the PennPET Explorer was not deliberately designed with axial inter-ring gaps, the images presented were acquired with data gaps between rings (Karp et al 2020). Because of the superior timing resolution of 250 ps, the reconstructed activity image has excellent image quality and uniformity independent of axial location. The superior timing resolution provides increased Fisher information, while the dramatically increased sensitivity from the long axial FOV scanner improves noise performance. Both superior timing resolution and dramatically increased sensitivity can be very beneficial to the joint reconstruction, which in turn motivated the development of practical joint reconstruction.

Conti (2011) showed that TOF information provides a more robust image reconstruction in the presence of inconsistent data. For joint reconstruction, the TOF information is utilized for the estimation of attenuation image, and the joint reconstruction can be less robust in the presence of inconsistent data. The residual errors in timing calibration, normalization and data corrections can introduce crosstalk artifacts between reconstructed activity and attenuation images (Salomon et al 2011, Rezaei et al 2012, Mehranian and Zaidi 2015). For scatter correction, TOF single scatter simulation (SSS) was used for scatter estimation (Watson 2007, Werner et al 2006). The extension of SSS to double scatter simulation for TOF PET scanners can provide more accurate scatter correction (Watson et al 2018). Our future work will study the influence of data corrections for scanners with long axial FOV to improve data consistency, and applying and evaluating the practical joint reconstruction with different VOI selections to clinical PET imaging applications.

## 6. Conclusion

In this paper, we presented a practical joint reconstruction of activity and attenuation with autonomous determination of scaling, and joint TOF scatter estimation from the reconstructed activity and attenuation images. The scaling factor is determined using a selected VOI with known attenuation in average. The scaling determination allows the proposed practical CT-less joint reconstruction to obtain a unique and faithful solution of activity and attenuation from TOF PET data. We demonstrated using both simulated and experimental data that the joint reconstruction with proper scaling can produce comparable results to the reference reconstruction with CT-based attenuation correction. The autonomous scaling with known attenuation in a VOI and joint TOF scatter estimation allow accurate joint reconstruction of activity and attenuation images when a CT scan is not used for radiation dose reduction.

## Footnotes

1 TOF scatter events contain attenuation information, and might actually improve attenuation reconstruction if properly utilized.

## Appendix A. Derivation of XMLTR (4)

Based on the log-likelihood function (2), TOF data do not provide additional attenuation information when there are no scattered events. We rewrite the log-likelihood function for non-TOF data with known activity image ***λ*** as

(A1) $$L\left(\mu \right)=y^{\text{T}}\log\left(pe^{-\chi \mu }+s\right)-1^{\text{T}}\left(pe^{-\chi \mu }+s\right),p=H\lambda .$$

Taking derivatives of (A1) with respect to ***λ***, we have gradient and Hessian matrix

(A2) $$\frac{\partial L}{\partial \mu }=\chi^{\text{T}}\left[pe^{-\chi \mu }\left(1-\frac{y}{pe^{-\chi \mu }+s}\right)\right],$$

(A3) $$\frac{\partial^{2}L}{\partial \mu \partial \mu^{\text{T}}}=-\chi^{\text{T}}\text{diag}\left[pe^{-\chi \mu }\left(1-\frac{ys}{{\left(pe^{-\chi \mu }+s\right)}^{2}}\right)\right]\chi .$$

The second-order Taylor series about ${\hat{\mu }}^{\left(k\right)}$ for the log-likelihood can be given by

(A4) $$L\left(\mu \right)\approx L\left({\hat{\mu }}^{\left(k\right)}\right)+\frac{\partial L}{\partial {\hat{\mu }}^{\left(k\right)}}\left(\mu -{\hat{\mu }}^{\left(k\right)}\right)+\frac{1}{2}{\left(\mu -{\hat{\mu }}^{\left(k\right)}\right)}^{\text{T}}\frac{\partial^{2}L}{\partial {\hat{\mu }}^{\left(k\right)}{\hat{\mu }}^{\left(k\right)\text{T}}}\left(\mu -{\hat{\mu }}^{\left(k\right)}\right).$$

Similar to the methods in (Nuyts et al 1998, Fessler et al 1997), we further approximate (A4) using a diagonal matrix by summing the rows of the Hessian matrix

(A5) $$L\left(\mu \right)\approx L\left({\hat{\mu }}^{\left(k\right)}\right)+\frac{\partial L}{\partial {\hat{\mu }}^{\left(k\right)}}\left(\mu -{\hat{\mu }}^{\left(k\right)}\right)\;+\frac{1}{2}{\left(\mu -{\hat{\mu }}^{\left(k\right)}\right)}^{\text{T}}\text{diag}\left[\frac{\partial^{2}L}{\partial {\hat{\mu }}^{\left(k\right)}\partial {\hat{\mu }}^{\left(k\right)\text{T}}}1\right]\left(\mu -{\hat{\mu }}^{\left(k\right)}\right)$$

To maximize (A5), we have zero gradient with respect to ***λ***,

(A6) $$\frac{\partial L}{\partial {\hat{\mu }}^{\left(k\right)}}+\text{diag}\left[\frac{\partial^{2}L}{\partial {\hat{\mu }}^{\left(k\right)}\partial {\hat{\mu }}^{\left(k\right)\text{T}}}1\right]\left(\mu -{\hat{\mu }}^{\left(k\right)}\right)=0$$

By directly solving (A6), we have

(A7) $$\mu ={\hat{\mu }}^{\left(k\right)}-\text{diag}\left[\frac{1}{\frac{\partial^{2}L}{\partial {\hat{\mu }}^{\left(k\right)}\partial {\hat{\mu }}^{\left(k\right)\text{T}}}1}\right]\frac{\partial L}{\partial {\hat{\mu }}^{\left(k\right)}}={\hat{\mu }}^{\left(k\right)}-\frac{\frac{\partial L}{\partial {\hat{\mu }}^{\left(k\right)}}}{\frac{\partial^{2}L}{\partial {\hat{\mu }}^{\left(k\right)}\partial {\hat{\mu }}^{\left(k\right)\text{T}}}1}$$

Putting (A2) and (A3) into (A7), we derive (4). When the measured data satisfying $ys/{\left(pe^{-\chi \mu }+s\right)}^{2}<1$, the Hessian matrix in (A3) is negative definite, and there exists a unique solution.
